# Integrative Network Pharmacology and Experimental Validation Reveal Multi-target Hepatoprotective Mechanisms of Bioactive Compounds from Xiao-Yao-San

**DOI:** 10.5812/ijpr-171015

**Published:** 2026-06-03

**Authors:** Zi-Yi Zheng, Jing-Yi Chen, Roy You Chen Quah, Guo-Hua Ding, Jing Chen

**Affiliations:** 1 School of Life Sciences, Zhejiang Chinese Medical University, Hangzhou, China; 2 College of Life Sciences, Nanjing Normal University, Nanjing, China; 3 Department of Biological Sciences, National University of Singapore, Singapore, Singapore; 4 College of Agriculture and Biotechnology, Lishui University, Lishui, China

**Keywords:** Hepatoprotection, Machine Learning, Multi-target Mechanisms, Network Pharmacology, Phytochemicals, Xiao-Yao-San

## Abstract

**Background:**

Xiao-Yao-San (XYS), a classical Traditional Chinese Medicine formula documented in the Chinese Pharmacopoeia, has long been used to treat hepatic disorders; however, its multi-target hepatoprotective mechanisms remain systematically uncharacterized.

**Objectives:**

This study aimed to elucidate the hepatoprotective mechanisms of XYS bioactive compounds against drug-induced liver injury (DILI) using an integrative network pharmacology and experimental approach.

**Methods:**

Network pharmacology was integrated with transcriptomic analysis and machine learning to identify hub targets. Molecular docking was used to predict interaction patterns and relative binding energies between compounds and target proteins. Hepatoprotective effects were validated in an acetaminophen (APAP)-induced hepatotoxicity model in HepG2 cells by measuring cytotoxicity markers and hub gene expression.

**Results:**

Network pharmacology identified 125 bioactive compounds with 121 targets overlapping DILI pathogenesis. Machine learning identified five hub genes: ALOX5, CYP1A2, CYP3A4, F3, and PDE5A. Molecular docking showed favorable binding affinities (-5.02 to -8.66 kcal/mol) for four compounds: Beta-sitosterol, kaempferol, luteolin, and quercetin. All four compounds attenuated APAP-induced hepatotoxicity and modulated hub gene expression, with beta-sitosterol showing the most comprehensive hepatoprotective profile.

**Conclusions:**

The bioactive compounds of XYS may contribute to hepatoprotection against DILI through multi-target regulation involving the ALOX5-LTB4 inflammatory axis, CYP-mediated metabolic regulation, F3-associated procoagulant signaling, and PDE5A/cGMP-related signaling.

## 1. Background

Drug-induced liver injury (DILI) represents a major clinical challenge and accounts for a substantial proportion of acute liver failure cases (1, 2). Based on pathogenic mechanisms, DILI is classified as dose-dependent, idiosyncratic, or indirect hepatotoxicity (3). Its clinical manifestations are highly heterogeneous, ranging from acute liver injury to chronic dysfunction, fibrosis, or life-threatening acute liver failure (4, 5). Mechanistically, DILI pathogenesis involves complex multidimensional processes, with organelle stress, cholestatic injury, and immune-mediated responses representing three major pathways that drive disease progression (6). Current DILI management faces 2 major challenges: diagnostic complexity due to difficult drug attribution and concurrent medication use, and the inadequacy of single-target therapeutic approaches for addressing the multifactorial pathological networks of DILI (7-9). These limitations highlight the urgent need for comprehensive multi-target therapeutic strategies.

Network pharmacology and bioinformatics are commonly used to investigate the multi-component and multi-target mechanisms of traditional medicines (10, 11). These approaches can link herbal compounds with disease-related targets and pathways and have been applied in studies of liver diseases and DILI (12-14). Xiao-Yao-San (XYS), first documented in the Taiping Huimin Heji Jufang compiled during the Song Dynasty (10th-13th century AD) and currently listed in the Chinese Pharmacopoeia (2020 Edition), is one of the most widely prescribed classical herbal formulas in Traditional Chinese Medicine. XYS comprises 8 medicinal herbs (Table S1) and has been shown to have anti-inflammatory and antioxidant properties and hepatoprotective activity in liver fibrosis, chronic liver dysfunction, and nonalcoholic fatty liver disease (15). However, the specific bioactive compounds, molecular targets, and multi-pathway regulatory mechanisms underlying its hepatoprotective effects in DILI remain incompletely characterized and warrant systematic mechanistic investigation.

## 2. Objectives

This study used network pharmacology analysis to predict candidate targets and potential active compounds of XYS in the prevention and treatment of DILI. Transcriptomic analysis, machine learning, and molecular docking were then integrated to further refine target screening. Finally, in vitro experimental validation was performed in an APAP-induced hepatotoxicity model in HepG2 cells to investigate the hepatoprotective effects of representative compounds and the corresponding target-related molecular changes. The results indicated that XYS exerts hepatoprotective effects through coordinated regulation of multiple pathological processes involved in DILI.

## 3. Methods

### 3.1. Drug and Disease Target Dataset Acquisition

The target acquisition workflow included compound screening, target prediction, target standardization, and disease target collection.

#### 3.1.1. Screening of XYS Bioactive Compounds

Bioactive compounds from the 8 herbs of XYS were retrieved from TCMSP. Compounds were screened using oral bioavailability (OB) ≥ 30% and drug-likeness (DL) ≥ 0.18, which are commonly used absorption, distribution, metabolism, and excretion criteria in TCMSP-based network pharmacology studies (16).

#### 3.1.2. Prediction and Standardization of XYS-Related Targets

For target prediction, the SMILES structures of the retained compounds were obtained from PubChem and submitted to SwissTargetPrediction (17). Potential targets were collected from both TCMSP and SwissTargetPrediction. For SwissTargetPrediction, only targets with probability ≥ 0.3 were retained to reduce low-confidence predictions (18). All compound-related targets were mapped to human UniProt identifiers, restricted to *Homo sapiens*, and deduplicated to generate the final XYS-related target dataset (19).

#### 3.1.3. Collection and Standardization of DILI-Related Targets

DILI-related targets were retrieved from GeneCards and OMIM using "drug induced liver injury" as the search term (20, 21). The retrieved targets were restricted to *Homo sapiens*, merged, and deduplicated to generate the final DILI-related target dataset.

### 3.2. Network Construction and Topological Analysis

Network analysis was performed to identify the potential therapeutic targets of XYS against DILI and evaluate their topological importance.

#### 3.2.1. Identification of Overlapping Targets and Protein-Protein Interaction Network Construction

Overlapping targets between the XYS-related and DILI-related datasets were identified by Venn analysis. These overlapping targets were imported into STRING (https://cn.string-db.org/) for protein-protein interaction (PPI) network construction. In STRING, the organism was set as *Homo sapiens*. The confidence score threshold was set to ≥ 0.7, and disconnected nodes were hidden to retain high-confidence interactions (22).

#### 3.2.2. Visualization and Topological Analysis of the Protein-Protein Interaction Network

The PPI network was visualized in Cytoscape v3.10.1, and degree centrality was calculated using CytoNCA v2.1.6 to rank node importance (23).

#### 3.2.3. Construction of the Herb-Compound-Target Network

A herb-compound-target tripartite network was also constructed in Cytoscape, with hub compounds identified by degree centrality.

### 3.3. Gene Expression Omnibus Dataset Acquisition and Transcriptome Analysis

Transcriptome analysis was used to provide expression-level evidence for the network-predicted targets.

#### 3.3.1. GEO Dataset Acquisition

The GSE93840 dataset was downloaded from the Gene Expression Omnibus (GEO) database and included 27 vehicle control samples and 27 drug-exposed samples (24).

#### 3.3.2. Differential Expression Analysis and Visualization

Differentially expressed genes (DEGs) were identified using limma v3.62.2. The cutoff values were |log2FC| ≥ 2 and FDR < 0.05 after Benjamini-Hochberg correction. These criteria were used to retain genes with robust expression changes while controlling the false discovery rate (25). The top 30 DEGs ranked by B-statistic were selected for visualization. Their expression patterns were displayed by Z-score-based hierarchical clustering.

#### 3.3.3. Integration of Network Pharmacology and Transcriptomic Evidence

To integrate network pharmacology with transcriptomic evidence, the candidate therapeutic targets obtained from the network analysis were intersected with the GSE93840-derived DEGs. This intersection generated 16 core target genes. Spearman correlation analysis was performed to evaluate co-expression relationships among these genes.

#### 3.3.4. Gene Ontology and Kyoto Encyclopedia of Genes and Genomes Enrichment Analysis

Gene Ontology (GO) and Kyoto Encyclopedia of Genes and Genomes (KEGG) enrichment analyses were performed using clusterProfiler v4.14.6 and enrichplot v1.26.6 (26).

### 3.4. Hub Gene Identification Using Machine Learning

Machine learning analysis was used to further prioritize hub genes from the 16 core target genes. The expression matrix of the 16 core target genes was used as the input data. Least absolute shrinkage and selection operator (LASSO) regression was performed using glmnet v4.1 - 8. Five-fold cross-validation was used to select the optimal λ. The random seed was set to 123 to improve reproducibility. Genes with non-zero coefficients were retained as LASSO-selected genes (27). Random Forest analysis was performed using randomForest v4.7.1.2. The number of trees was set to 500. The model with the lowest out-of-bag error was selected. Genes with importance scores > 1.5 were retained as Random Forest-selected genes. This threshold was used as an empirical feature-selection cutoff (28). Genes selected by both LASSO regression and Random Forest analysis were defined as hub genes. This overlap-based strategy was used to improve the robustness of hub gene selection.

### 3.5. Molecular Docking

Molecular docking was performed to evaluate the structural plausibility of interactions between representative XYS compounds and hub targets.

#### 3.5.1. Receptor and Ligand Preparation

The three-dimensional crystal structures of the target proteins were obtained from the Protein Data Bank database (https://www.rcsb.org/) (29). Water molecules, original ligands, and other heteroatoms were removed using PyMOL v3.1. Receptor and ligand preparation was performed using AutoDock Tools v1.5.6, including hydrogen addition, charge assignment, and grid box setup.

#### 3.5.2. Docking Procedure

Blind docking was carried out using AutoDock 4.2.6 with the Lamarckian genetic algorithm (30). Binding energies ≤ -5.0 kcal/mol were regarded as favorable predicted binding affinities (31).

#### 3.5.3. Binding Mode Visualization

For each docking pair, the conformation with the lowest predicted binding energy was selected as the representative binding mode and visualized using PyMOL v3.1. Protein IDs and docking grid box parameters are listed in Table S5.

### 3.6. Cell-Based Experimental Validation

Based on the predicted hub targets and selected compounds, an APAP-induced hepatotoxicity model was established in HepG2 cells for experimental validation.

#### 3.6.1. Cell Culture

HepG2 cells were purchased from Shanghai Zhong Qiao Xin Zhou Biotechnology Co., Ltd., and cultured in DMEM supplemented with 10% fetal bovine serum, 100 U/mL penicillin, and 100 μg/mL streptomycin. Cells were maintained in a humidified incubator at 37 °C with 5% CO_2_.

#### 3.6.2. Preliminary CCK-8 Concentration Screening

The final concentrations of APAP and test compounds were determined by preliminary CCK-8 assays. HepG2 cells were exposed to serial concentrations of APAP or individual compounds for 24 hours, followed by incubation with CCK-8 reagent. Absorbance was measured at 450 nm, and cell viability was calculated relative to the vehicle control group. The APAP concentration was selected based on the IC_50_ value, whereas compound concentrations were selected according to the safe concentration, defined as the highest concentration maintaining cell viability ≥ 90%. Detailed procedures for concentration screening and calculation of the APAP IC_50_ and compound C_safe_ values are provided in Supplementary Methods S2.

#### 3.6.3. APAP-Induced Hepatotoxicity Model

Based on the preliminary CCK-8 screening results, HepG2 cells were treated with 30.8 mM APAP for 24 hours to establish the hepatotoxicity model.

#### 3.6.4. Compound Treatment

Beta-sitosterol, kaempferol, luteolin, and quercetin were dissolved in DMSO and applied at their non-cytotoxic concentrations of 12.2, 6.0, 24.5, and 13.0 μM, respectively. The final DMSO concentration in the culture medium was kept below 0.1% in all groups. Cells in the vehicle control, APAP model, and compound co-treatment groups received equivalent concentrations of DMSO. Cells were divided into the vehicle control group, APAP model group, and APAP plus individual compound co-treatment groups. Each compound was added simultaneously with APAP, followed by co-incubation for 24 hours.

#### 3.6.5. Cytotoxicity and Hepatocellular Injury Assays

Cell viability was assessed using the CCK-8 assay. Membrane damage and hepatocellular injury were evaluated by measuring LDH release, ALT levels, and AST levels. Detailed information on the CCK-8 kit, LDH assay kit, ALT assay kit, AST assay kit, manufacturers, and catalog numbers is provided in Methods S1 in the Supplementary File.

#### 3.6.6. Reverse Transcription Quantitative PCR Assay

The mRNA expression levels of ALOX5, CYP1A2, CYP3A4, F3, and PDE5A were measured by reverse transcription quantitative PCR. GAPDH was used as the internal reference gene, and relative gene expression was calculated using the 2^-ΔΔCt^ method. Detailed information on the RT-qPCR reagents, manufacturers, and catalog numbers is provided in Methods S1 in the Supplementary File. Primer sequences are listed in Table S2.

#### 3.6.7. ELISA Assays

Related proteins or functional mediators, including LTB4, CYP1A2, CYP3A4, F3, and cGMP, were measured using ELISA kits. Detailed information on the ELISA kits, manufacturers, and catalog numbers is provided in Supplementary Methods S1.

### 3.7. Statistical Analysis

All computational analyses were performed using R v4.4.3. The chi-squared (χ^2^) test was used to compare proportions of up- and down-regulated DEGs, and Spearman rank correlation was used to assess co-expression patterns among core target genes. For experimental validation, normality and homogeneity of variance were tested using the Shapiro-Wilk and Levene tests, respectively. One-way ANOVA with Tukey HSD post hoc test was used for group comparisons. Data were expressed as mean ± standard deviation (SD) from 3 independently repeated tests. Statistical significance was defined as P < 0.05.

## 4. Results

### 4.1. Target Identification and Network Construction

A total of 125 bioactive compounds were identified from XYS through TCMSP database screening. Eight compounds (beta-sitosterol, kaempferol, sitosterol, stigmasterol, isorhamnetin, mairin, naringenin, and quercetin) were shared across multiple herbs, with the remaining compounds showing herb-specific distribution (Figure S1 and Table S3 in the Supplementary File). After deduplication and normalization of 2754 putative targets from TCMSP and Swiss Target Prediction, 351 XYS-associated proteins were retained. GeneCards and OMIM queries yielded 1134 DILI-related target genes after filtering and deduplication.

Intersection analysis identified 121 overlapping genes between XYS targets and DILI-related targets (Figure 1A), which were used to construct a PPI network of 121 nodes and 4554 edges via STRING (Figure 1B). A herb-compound-target tripartite network of 8 herbs, 125 compounds, and 351 target genes (485 nodes and 2891 edges) was constructed, with degree centrality analysis identifying quercetin, with a degree centrality of 458; kaempferol, with a degree centrality of 437; beta-sitosterol, with a degree centrality of 132; and luteolin, with a degree centrality of 113, as the top hub compounds (Figure 1C-D).

**Figure 1.**
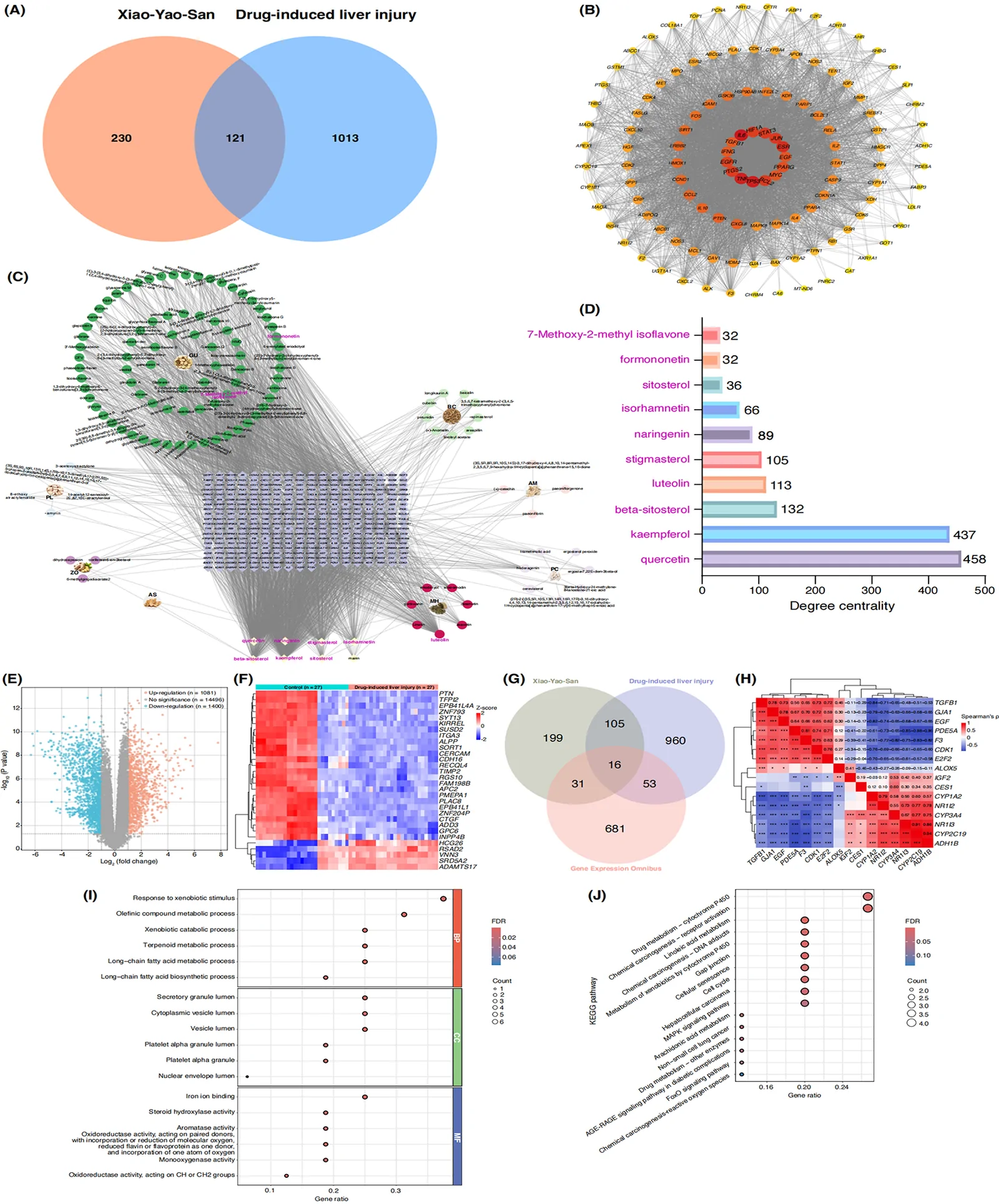
Integrated network pharmacology and transcriptomic analysis for identifying Xiao-Yao-San intervention targets in drug-induced liver injury. (A) Venn diagram showing 121 overlapping genes between XYS target proteins (n = 351) and DILI-associated genes (n = 1134). (B) PPI network of the 121 overlapping target proteins. Node size and color intensity indicate degree centrality, with larger and redder nodes indicating higher degree values and more pivotal roles in the network. (C) Herb-compound-target tripartite network showing relationships between XYS herbs (orange squares), compounds (circles/diamonds), and target proteins (purple diamonds) (Abbreviations: AM, *Atractylodes macrocephala* Koidz; AS, *Angelica sinensis* (Oliv.) Diels.; BC, *Bupleurum chinense* DC.; GU, *Glycyrrhiza uralensis* Fisch.; MH, *Mentha haplocalyx* Briq.; PC, *Poria cocos* (Schw.) Wolf.; PL, *Paeonia lactiflora* Pall.; and ZO, *Zingiber officinale* Rosc). (D) Top 10 compounds ranked by degree centrality, with quercetin showing the highest connectivity. (E) Volcano plot of DEGs in the GSE93840 dataset comparing control and DILI groups. Red dots indicate up-regulated genes (log2FC > 2, FDR < 0.05), blue dots indicate down-regulated genes (log2FC < -2, FDR < 0.05), and gray dots represent non-significant genes. (F) Heatmap of the top 30 DEGs between control and DILI groups, with hierarchical clustering. (G) Venn diagram showing 16 overlapping genes between XYS potential targets (121 genes) and DILI-related DEGs (781 genes) from the GEO database. (H) Spearman correlation matrix of the 16 core target genes. Red indicates positive correlations, while blue indicates negative correlations. (I) Top 6 GO terms enriched in the 16 core target genes, showing biological processes, cellular components, and molecular functions. (J) Sixteen KEGG pathways. Dot size represents gene count; color intensity indicates FDR-value significance.

### 4.2. Differential Expression Analysis and Core Target Gene Identification

Analysis of GSE93840 identified 781 DEGs from 16 978 genes, with down-regulated genes predominating (χ^2^ test: χ^2^ = 40.11, df = 1, P < 0.001) (Figure 1E). The top 30 DEGs showed distinct expression patterns between groups (Figure 1F). Intersection of the 121 network pharmacology targets with DEGs identified 16 core DILI-related target genes (Figure 1G, Table S4 in the Supplementary File). TGFB1, GJA1, CDK1, EGF, and F3 showed strong positive correlations (r > 0.65), while CYP1A2, CYP2C19, NR1I2, and NR1I3 exhibited strong negative correlations (r < -0.6) (Figure 1H). GO and KEGG enrichment analyses were performed on the 16 core target genes to clarify their functional significance. These analyses highlighted xenobiotic metabolism, oxidoreductase activity, and drug metabolism-cytochrome P450/ROS/FoxO pathways (Figure 1I-J), suggesting modulation of drug metabolism, oxidative stress, and cell cycle control.

### 4.3. Machine Learning-Based Hub Gene Identification

Two machine learning approaches were applied for exploratory feature selection of hub genes associated with DILI pathogenesis. LASSO regression identified 8 feature genes at the optimal regularization parameter λ, as evidenced by the characteristic U-shaped curve of mean cross-validation error (Figure 2A) and coefficient shrinkage paths (Figure 2B). The RF model achieved stable out-of-bag error after approximately 150 trees (Figure 2C) and identified 8 genes with variable importance scores exceeding 1.5 (Figure 2D). Venn diagram analysis revealed 5 overlapping genes between the 2 algorithms: ALOX5, CYP1A2, CYP3A4, F3, and PDE5A (Figure 2E). The convergence of 2 independent machine learning algorithms on these 5 genes provides additional support for prioritizing them for subsequent experimental validation.

**Figure 2.**
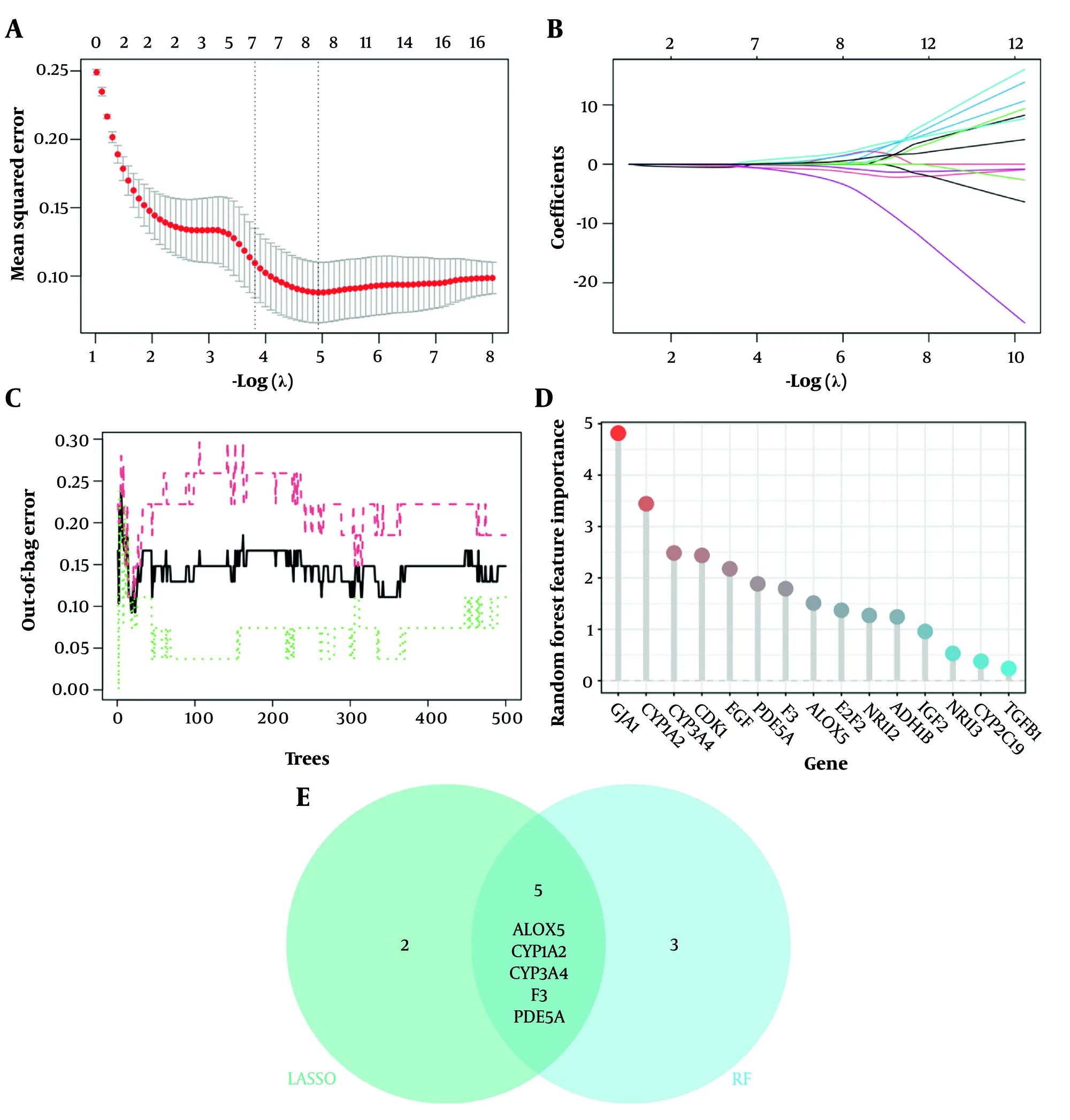
Machine learning-based hub gene identification. (A) Least absolute shrinkage and selection operator cross-validation error curve with optimal lambda (λ) parameter selection. Numbers above the plot represent the number of non-zero coefficients at each λ value. (B) LASSO coefficient shrinkage paths showing the variable selection process across different λ values. Each colored line represents the trajectory of 1 gene coefficient. Numbers above the plot indicate the number of non-zero coefficients remaining at each λ value. (C) Random Forest out-of-bag error curve across different numbers of decision trees. (D) Random Forest variable importance plot ranking genes by their importance scores. (E) Venn diagram showing 5 overlapping hub genes (CYP1A2, CYP3A4, PDE5A, F3, and ALOX5) identified by both LASSO and Random Forest algorithms.

### 4.4. Molecular Docking

Molecular docking of 4 bioactive compounds against 5 hub targets yielded predicted binding energies of -5.02 to -8.66 kcal/mol across 20 complexes (Table S5), indicating generally favorable predicted binding affinities. Among them, the CYP3A4-beta-sitosterol complex exhibited the lowest predicted binding energy (-8.66 kcal/mol), followed by CYP1A2-beta-sitosterol (-7.92 kcal/mol) and CYP1A2-quercetin (-7.24 kcal/mol). Key interactions in the 8 top-ranked complexes included hydrogen bonding and hydrophobic contacts (Figure 3A-H). Detailed visualization of all 20 docked complexes is provided in Figure S2. These results provide structural-level support for potential interactions between the active compounds of XYS and DILI-related hub proteins. However, predicted binding affinity alone does not establish direct binding or biological relevance, necessitating experimental validation to confirm functional relevance.

**Figure 3.**
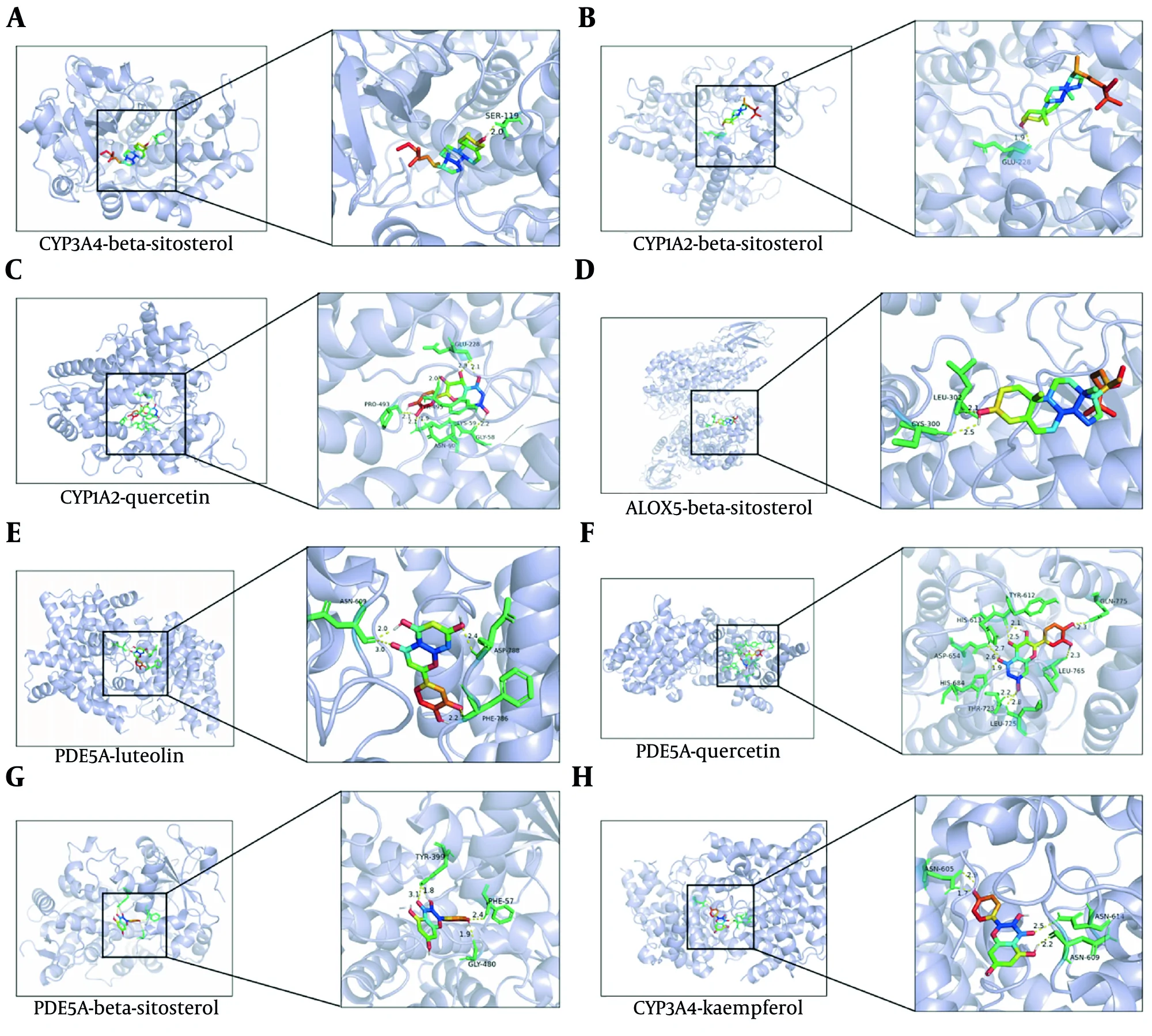
Molecular docking analysis between top bioactive compounds identified from Xiao-Yao-San and hub proteins associated with drug-induced liver injury. Representative docking conformations with the lowest predicted binding energies are shown for 8 protein-ligand pairs: (A) CYP3A4-beta-sitosterol (-8.66 kcal/mol), (B) CYP1A2-beta-sitosterol (-7.92 kcal/mol), (C) CYP1A2-quercetin (-7.24 kcal/mol), (D) ALOX5-beta-sitosterol (-7.18 kcal/mol), (E) PDE5A-luteolin (-7.11 kcal/mol), (F) PDE5A-quercetin (-7.02 kcal/mol), (G) PDE5A-beta-sitosterol (-6.93 kcal/mol), and (H) CYP3A4-kaempferol (-6.65 kcal/mol). For each panel, the left image shows the overall protein-ligand binding mode with protein structures rendered as light purple cartoons and ligands as sticks. The right image displays an enlarged view of the binding pocket, highlighting key interacting residues (green sticks), ligands (colored sticks), and hydrogen bonds (yellow dashed lines).

### 4.5. Cell-Based Experimental Validation

APAP treatment significantly decreased cell viability and elevated LDH release, ALT, and AST levels compared with the vehicle control (one-way ANOVA: All P < 0.001) (Figure 4A-D), confirming the establishment of an APAP-induced hepatotoxicity model in HepG2 cells. When beta-sitosterol, kaempferol, luteolin, and quercetin were individually co-treated with APAP, cell viability in all co-treatment groups was lower than that in the vehicle group but higher than that in the APAP group (Figure 4A). All 4 compounds also attenuated APAP-induced increases in LDH release and ALT levels (Figure 4B-C), with beta-sitosterol showing the strongest overall effect. In contrast, quercetin showed the greatest reduction in AST, whereas kaempferol failed to significantly reduce AST compared with the APAP group (Figure 4D).

**Figure 4.**
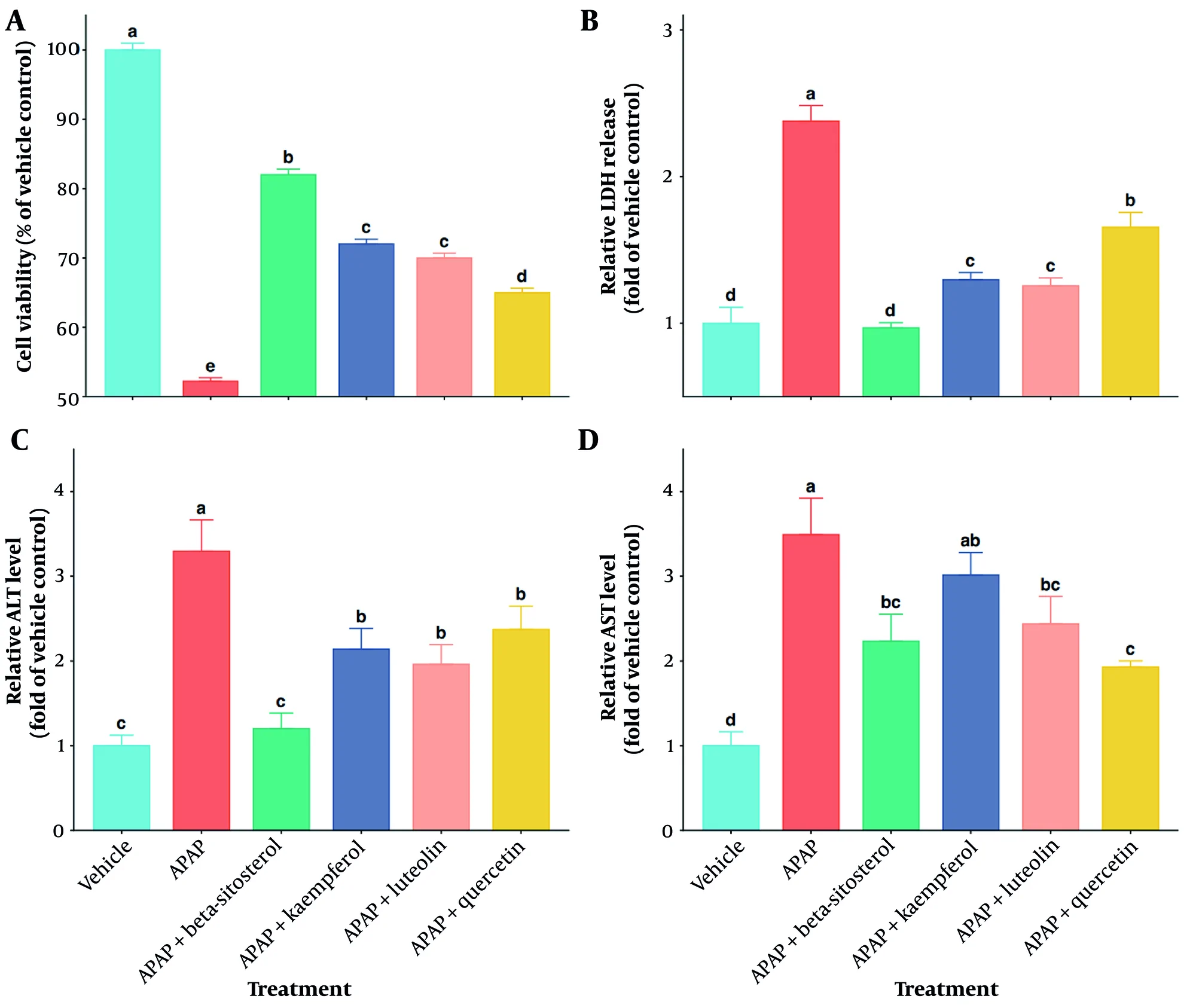
Cytoprotective effects of Xiao-Yao-San bioactive compounds in an in vitro drug-induced liver injury model. (A) Cell viability determined by the Cell Counting Kit-8 assay. (B) Relative lactate dehydrogenase release. (C) Relative alanine aminotransferase levels. (D) Relative aspartate aminotransferase levels. Data are presented as mean ± SD from 3 independent biological replicates (n = 3). Bars with different letters indicate significant differences among groups (one-way ANOVA followed by Tukey HSD test, P < 0.05); bars sharing at least 1 letter are not significantly different.

The expression levels of 5 hub target genes (ALOX5, CYP1A2, CYP3A4, F3, and PDE5A) and their corresponding protein or functional markers differed among the 6 treatment groups (one-way ANOVA: all P < 0.001). Compared with the vehicle group, APAP treatment up-regulated the expression of ALOX5, F3, and PDE5A, while down-regulating CYP1A2 and CYP3A4. The corresponding proteins or functional markers showed changes that were partially consistent with their respective mRNA expression patterns (Figure 5A-J).

**Figure 5.**
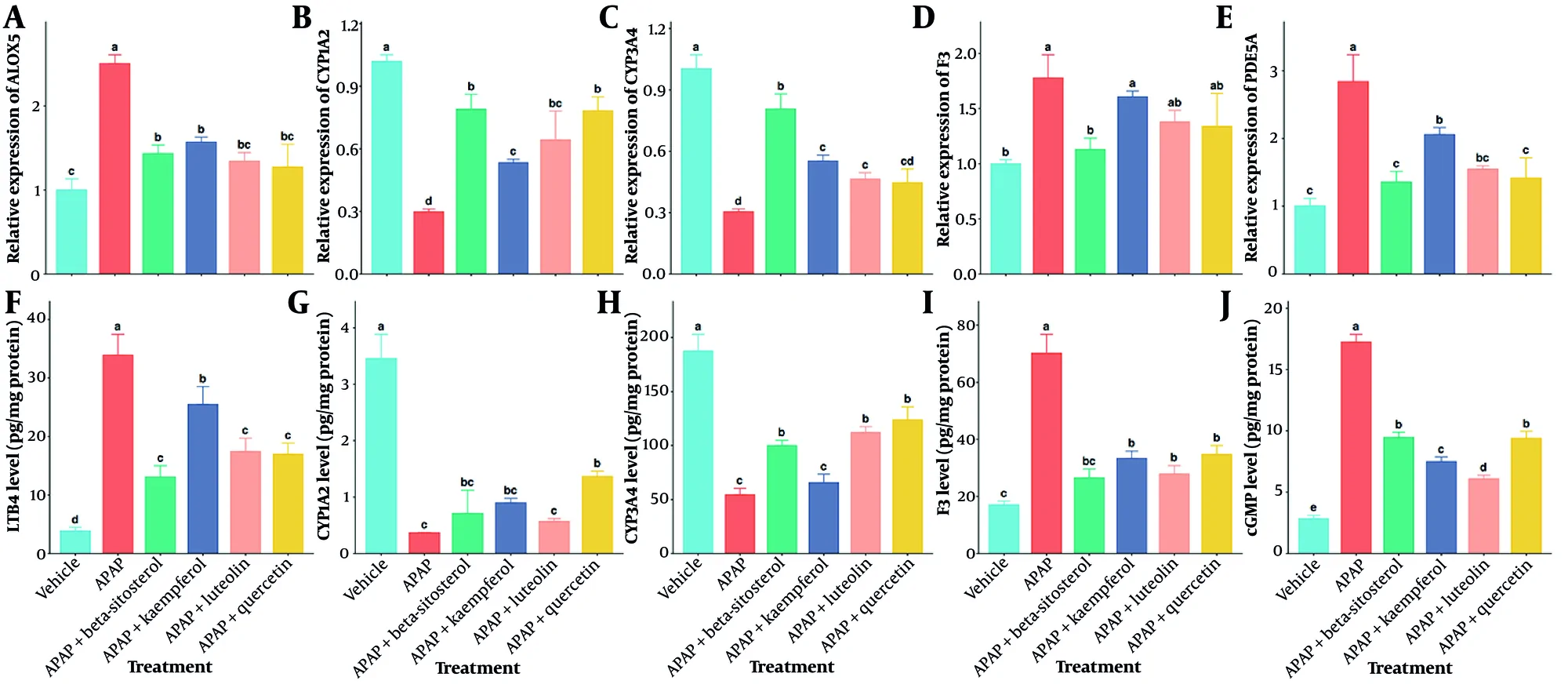
Effects of Xiao-Yao-San bioactive compounds on hub gene expression and related functional markers in the APAP-induced hepatotoxicity model. (A-E) Relative mRNA expression levels of ALOX5, CYP1A2, CYP3A4, F3, and PDE5A. (F-J) Levels of LTB4, CYP1A2, CYP3A4, F3, and cGMP. Data are presented as mean ± SD from 3 independent biological replicates (n = 3). Bars with different letters indicate significant differences among groups (one-way ANOVA followed by Tukey HSD test, P < 0.05); bars sharing at least 1 letter are not significantly different.

All 4 compounds suppressed ALOX5 upregulation and reduced LTB4 accumulation (Figure 5A and F). All 4 compounds partially restored CYP1A2 and CYP3A4 expression at the mRNA level or CYP1A2 and CYP3A4 protein levels, although compound-specific discordances were observed between transcriptional and translational responses (Figure 5B, C, G, and H). Furthermore, transcriptional and translational changes in F3 were not entirely concordant: Only beta-sitosterol significantly suppressed F3 upregulation at the mRNA level, whereas all 4 compounds reduced APAP-induced F3 protein elevation (Figure 5D and I). Compared with the APAP group, all 4 compounds also suppressed aberrant PDE5A upregulation and reduced accumulation of its functional marker, cGMP (Figure 5E and J). Taken together, these results indicate that the 4 representative compounds partially alleviated APAP-induced hepatotoxicity and differentially modulated hub gene-related molecular changes in HepG2 cells.

## 5. Discussion

DILI is a clinically heterogeneous disorder with complex and multifactorial pathogenesis. The prevention and treatment of DILI therefore remain urgent clinical needs (32). Previous studies have highlighted the hepatoprotective potential of natural bioactive compounds in liver injury (33). In the present study, network pharmacology, transcriptomic data, machine learning, and molecular docking were integrated to preliminarily identify the core compounds, hub targets, and potential mechanisms through which XYS may protect against DILI. Subsequent cell-based experimental validation provided further support for these predictions, suggesting that XYS may have potential application value in DILI intervention. This finding is broadly consistent with recent evidence showing that plant-derived extracts can alleviate chemically induced hepatotoxicity in HepG2 cells. For example, dandelion hydroalcoholic extract has been reported to reduce carbon tetrachloride-induced cytotoxicity and apoptosis by modulating Bax/Bcl-2 expression (34).

A notable strength of the present study is its integrative design. Rather than relying solely on compound screening or bioinformatic prediction, computational prioritization was combined with experimental validation, thereby narrowing a broad candidate space into biologically interpretable compound-target relationships. Four putative core compounds, namely beta-sitosterol, kaempferol, luteolin, and quercetin, were prioritized from XYS. Previous studies have suggested that these compounds have hepatoprotective, anti-inflammatory, and antioxidant activities (35-38).

Among the validated modules, the ALOX5-related inflammatory axis was the most consistently supported. ALOX5, a rate-limiting enzyme in leukotriene biosynthesis, is closely associated with inflammatory amplification and oxidative stress (39, 40). All 4 compounds suppressed APAP-induced ALOX5 upregulation and reduced LTB4 accumulation, suggesting that modulation of the ALOX5-LTB4 inflammatory axis may represent a shared protective mechanism.

By contrast, support for the CYP-related metabolic module was more limited and should be interpreted with caution. Previous studies indicate that a substantial proportion of idiosyncratic DILI cases involve cytochrome P450 metabolism, with CYP1A2 and CYP3A4 serving as core metabolic hubs that drive drug bioactivation, generate toxic intermediates, and ultimately induce liver injury (41). Although all 4 compounds partially restored CYP1A2 and CYP3A4 mRNA expression or protein levels, CYP-related readouts should be interpreted as auxiliary metabolic indicators pending validation in more metabolically competent systems, given the intrinsically low basal CYP expression and limited metabolic capacity of HepG2 cells and the observed discordance between transcriptional and translational responses.

The F3-associated module also merits attention. F3 initiates the coagulation cascade and contributes to procoagulant states in chronic liver disease and DILI models, linking hemostatic activation to disease progression (42, 43). Only beta-sitosterol significantly suppressed F3 upregulation at the mRNA level, whereas all 4 compounds reduced the elevation of F3 protein levels, suggesting that regulation of the F3 axis may occur at multiple levels. However, coagulation-related functional endpoints were not directly measured in the present study. Therefore, the current data support only modulation of the F3-associated module by these compounds, and any effect on coagulation function remains speculative and requires further experimental validation. Hepatic microcirculatory dysfunction is considered a critical pathological process in hepatotoxic injury (44).

In addition, the PDE5A/cGMP axis may represent a relatively exploratory but potentially important mechanistic dimension of XYS-mediated hepatoprotection. As an important regulator of vascular tone, PDE5A may participate in the regulation of hepatic vascular tone by modulating cGMP signaling (45, 46). Under APAP-induced conditions, PDE5A/cGMP-related molecular changes were also observed, raising the possibility that these compounds participate in hepatoprotection partly through modulation of the PDE5A/cGMP signaling axis.

Differences among the 4 compounds also deserve consideration. Beta-sitosterol exhibited the broadest and most balanced regulatory profile across the tested readouts, including cytoprotective effects, F3-associated regulation, and selected indicators related to metabolism and signaling. Quercetin showed relatively stronger effects on ALOX5-related mRNA changes and AST reduction, whereas luteolin was more prominently associated with cGMP-related signaling regulation. In contrast, kaempferol showed comparatively weaker overall effects in this model despite prior reports of hepatoprotection. These differences may reflect variation in experimental context, dominant injury mechanisms, compound concentration, or model-specific limitations, especially the restricted metabolic competence of HepG2 cells. More importantly, they suggest that the contributions of individual XYS-derived compounds may be distinct yet complementary.

Overall, the combined computational and experimental findings support the notion that XYS exerts hepatoprotective effects through multi-compound, multi-target regulation across several pathological modules involved in DILI rather than through a single mechanism, although further in-depth experimental validation is still required.

### 5.1. Limitations and Future Perspectives

While this study systematically explored the multi-compound mechanisms underlying the hepatoprotective effects of XYS, several limitations should be acknowledged. First, because the computational workflow integrated predicted targets, curated disease-gene sets, transcriptomic signals, and machine learning-based prioritization, potential bias and circularity cannot be fully excluded. Hub gene identification based on public databases and transcriptomic data may be influenced by technical variability, and transcriptional changes do not necessarily reflect protein abundance or functional activity (47, 48). Although multiple analytical layers, molecular docking, and in vitro validation were used to improve robustness, some degree of selection bias may still remain. Second, the APAP-induced hepatotoxicity model in HepG2 cells used for experimental validation mainly reflects a reactive intrinsic hepatotoxicity paradigm. Therefore, caution is needed when extrapolating these findings to other, more complex forms of DILI, particularly idiosyncratic DILI. In addition, HepG2 cells exhibit relatively low CYP expression and limited metabolic capacity in the APAP-induced hepatotoxicity model, which may restrict evaluation of the metabolism-related protective effects of XYS (32, 49). More physiologically relevant models, such as primary human hepatocytes, are therefore needed in future studies. Moreover, 3D culture systems may better preserve key hepatic functions, including the expression of drug-metabolizing enzymes, over extended periods, thereby providing a more suitable platform for long-term and repeated-dose hepatotoxicity assessment (50). In addition, the APAP-induced hepatotoxicity model in HepG2 cells lacks non-parenchymal and immune cell components of the hepatic microenvironment and therefore cannot adequately recapitulate the more complex inflammatory or immune-associated processes involved in DILI. Future studies should incorporate more physiologically relevant co-culture systems, such as hepatocyte-nonparenchymal cell co-culture models, to better capture heterogeneous cell-cell interactions and the inflammatory hepatic microenvironment (51). In addition, in vivo models and multi-omics approaches should be integrated to further evaluate the applicability and mechanistic basis of XYS in broader DILI contexts.

### 5.2. Conclusions

This study integrated network pharmacology, transcriptomic analysis, machine learning, molecular docking, and cell-based validation to identify 4 representative candidate compounds from XYS, namely beta-sitosterol, kaempferol, luteolin, and quercetin, as well as 5 key genes that may be involved in its hepatoprotective effects against DILI: ALOX5, CYP1A2, CYP3A4, F3, and PDE5A. Experimental results support the possibility that XYS exerts hepatoprotective effects through coordinated regulation of multiple pathological processes, including inflammatory activation, metabolic disturbance, coagulation dysregulation, and signaling imbalance, although the individual mechanistic axes still require further investigation. In addition, the broader applicability of these findings to DILI biology requires further refinement and validation through both in vitro and in vivo studies.

ijpr-25-1-171015-s001.pdf

## Data Availability

The public transcriptomic dataset analyzed in this study, GSE93840, is available from the Gene Expression Omnibus (GEO) database (https://www.ncbi.nlm.nih.gov/geo/). Supplementary Methods and key study-generated result tables are provided in the Supplementary Files. The dataset supporting the findings of this study is also publicly available on Figshare at https://doi.org/10.6084/m9.figshare.30812324.
