# Supplementary material for: Integrative Network Pharmacology and Experimental Validation Reveal Multi-target Hepatoprotective Mechanisms of Bioactive Compounds from Xiao-Yao-San
Source: Iran J Pharm Res. 2026 Jun 3;25(1):e171015. doi: 10.5812/ijpr-171015 (PMC13769049; doi:10.5812/ijpr-171015)
Supplement: Supplementary file 1 [file ijpr-25-1-171015-s001.pdf]

Supplementary Methods

S1 Chemicals and reagents

Acetaminophen (APAP, CAS: 103-90-2, PubChem CID: 1983, ≥98% purity) was purchased from Sangon Biotech (Shanghai, China). beta-sitosterol (CAS: 83-46-5, PubChem CID: 222284), luteolin (CAS: 491-70-3, PubChem CID: 5280445, ≥98% purity), kaempferol (CAS: 520-18-3, PubChem CID: 5280863, ≥95% purity), and quercetin (CAS: 117-39-5, PubChem CID: 5280343, ≥98% purity) were purchased from Anpel Laboratory (Shanghai, China) as BBI reference materials. Dulbecco's Modified Eagle Medium (DMEM), fetal bovine serum (FBS), penicillin-streptomycin solution, and trypsin-EDTA were purchased from Gibco (Thermo Fisher Scientific, USA). Dimethyl sulfoxide (DMSO, cell culture grade, ≥99.7%) was obtained from Sigma-Aldrich (St. Louis, MO, USA). Cell Counting Kit-8 (CCK-8, C0042), Lactate Dehydrogenase (LDH) Cytotoxicity Assay Kit (C0017), Alanine Aminotransferase (ALT) Assay Kit (P2711M), Aspartate Aminotransferase (AST) Assay Kit (P2715M), RIPA Lysis Buffer (P0013B), and BCA Protein Assay Kit (P0010) were purchased from Beyotime Biotechnology (Shanghai, China). RNA extraction reagent (G3013) was obtained from Thermo Fisher Scientific. SweScript All-in-One RT SuperMix for qPCR (with gDNA Remover, G3337) and 2× Universal Blue SYBR Green qPCR Master Mix (G3326) were purchased from TIANGEN Biotech (Beijing, China). Enzyme-linked immunosorbent assay (ELISA) kits for cyclic guanosine monophosphate (cGMP, E-EL-0083) and tissue factor (F3, E-EL-H0040) were obtained from Elabscience (Elabscience Biotechnology Co. Ltd., Wuhan, China). Leukotriene B4 (LTB4) ELISA Kit (502390) was purchased from Cayman Chemical (Ann Arbor, MI, USA). Cytochrome P450 1A2 (CYP1A2) (EH0777), and cytochrome P450 3A4 (CYP3A4) (EM0971) protein quantification ELISA kits were obtained from FineTest (Wuhan Fine Biotech Co. Ltd., Wuhan, China).

S2 Concentration screening and calculation of IC_{50} and C_{safe}

Concentration screening was performed to evaluate the cytotoxicity of APAP and the four active compounds, including beta-sitosterol, kaempferol, luteolin, and quercetin, in HepG2 cells. Cells were exposed to serial concentrations of APAP or individual compounds for 24 h after attachment. Cell viability was then assessed using the CCK-8 assay. 10 μL of CCK-8 reagent was added to each well, and the plates were incubated for 1.5 h. Absorbance was measured at 450 nm using a microplate reader (Infinite 200 PRO, Tecan, Austria). Cell viability was calculated as a percentage relative to the vehicle control after blank correction. The APAP IC_{50} was calculated using four-parameter logistic regression

with the drc R package. The safe concentration (C_{safe}) of each active compound was defined as the highest concentration maintaining cell viability ≥90% and was estimated by linear interpolation between adjacent experimental data points using the approx() function in R.

Table S1. Abbreviations of herbal medicines in the Xiao-Yao-San (XYS).

Abbreviation	Latin name	Chinese name	English name
AM	*Atractylodes macrocephala* Koidz.	白术	Atractylodes
AS	*Angelica sinensis* (Oliv.) Diels	当归	Angelica
BC	*Bupleurum chinense* DC.	柴胡	Bupleurum
GU	*Glycyrrhiza uralensis* Fisch.	甘草	Licorice
MH	*Mentha haplocalyx* Briq.	薄荷	Mint
PC	*Poria cocos* (Schw.) Wolf	茯苓	Hoelen
PL	*Paeonia lactiflora* Pall.	白芍	White peony
ZO	*Zingiber officinale* Rosc.	生姜	Ginger

Table S2. Primer sequences for quantitative real-time PCR.

Gene symbol (accession number)	Protein	Primer (5'-3')	Tm (°C)	Amplicon size
ALOX5 (NM_000698.5)	Arachidonate 5-lipoxygenase	F: TGGCCTACTTCTACCTGGTCAC<br>R: GGCAATGGGAACAGGTCTTG	56	149
CYP1A2 (NM_000761.5)	Cytochrome P450 family 1 subfamily A member 2	F: TGGAGACCTTCCGTTACCAG<br>R: GAACACGGTTGGCAGTCTTC	55	148
CYP3A4 (NM_017460.6)	Cytochrome P450 family 3 subfamily A member 4	F: CATTCCCTATAATACTTGGCCTTTT<br>R: GTCCCACCATCCTTCTTTATTTC	57	139
F3 (NM_001993.5)	Tissue factor	F: TGTCAACATCAAGCAGCAGG<br>R: CCAGGATGGCAGTGAAGATC	58	140
PDE5A (NM_001083.4)	Phosphodiesterase type 5A	F: CCACCAGATCCTGAAGACCA<br>R: TCCAGGTCATTGAGGTCAAG	58	155
GAPDH (NM_002046.7)	Glyceraldehyde-3-phosphate dehydrogenase	F: GCCTCCTGCACCACCAACTG<br>R: CCATCACGCCACAGTTTCCC	57	149

Table S3. Distribution of bioactive compounds in the Xiao-Yao-San (XYS) screened from TCMSP database (OB ≥ 30%, DL ≥ 0.18). Herb codes are defined in Table S1.

Bioactive compound	Herb code	Count
Beta-sitosterol	AS, PL, ZO	3
Stigmasterol	AS, BC, ZO	3
Kaempferol	BC, GU, PL	3
Sitosterol	GU, MH, PL	3
Isorhamnetin	BC, GU	2
Quercetin	BC, GU	2
Naringenin	GU, MH	2
Mairin	GU, PL	2
(3S,8S,9S,10R,13R,14S,17R)-10,13-dimethyl-17-[(2R,5S)-5-propan-2-yl	AM	1

Bioactive compound	Herb code	Count
octan-2-yl]-2,3,4,7,8,9,11,12,14,15,16,17-dodecahydro-1H-cyclopenta[a] phenanthren-3-ol		
14-acetyl-12-senecioyl-2E,8Z,10E-atractylentriol	AM	1
3beta-acetoxyatractylone	AM	1
8beta-ethoxy atractylenolide III	AM	1
Alpha-Amyrin	AM	1
Linoleyl acetate	BC	1
3,5,6,7-tetramethoxy-2-(3,4,5-trimethoxyphenyl)chromone	BC	1
Cubebin	BC	1
Petunidin	BC	1
(+)-Anomalin	BC	1
Baicalin	BC	1
Longikaurin A	BC	1
Alpha-spinasterol	BC	1
Areapillin	BC	1
Dehydroglyasperins C	GU	1
Xambioona	GU	1
Phaseol	GU	1
Odoratin	GU	1
Licoagroisoflavone	GU	1
Glycyrrhiza flavonol A	GU	1
Glyasperins M	GU	1
Licoagrocarpin	GU	1
Gancaonin H	GU	1
Gancaonin G	GU	1
Gadelaidic acid	GU	1
8-prenylated eriodictyol	GU	1
7-Acetoxy-2-methylisoflavone	GU	1
7,2',4'-trihydroxy－5-methoxy-3－arylcoumarin	GU	1
6-prenylated eriodictyol	GU	1
Kanzonol F	GU	1
Icos-5-enoic acid	GU	1
Inflacoumarin A	GU	1
2-[(3R)-8,8-dimethyl-3,4-dihydro-2H-pyrano[6,5-f]chromen-3-yl]-5-methoxyphenol	GU	1
3'-Methoxyglabridin	GU	1
3'-Hydroxy-4'-O-Methylglabridin	GU	1
Quercetin der.	GU	1

Bioactive compound	Herb code	Count
1-Methoxyphaseollidin	GU	1
HMO	GU	1
Isolicoflavonol	GU	1
Isoglycyrol	GU	1
(2S)-7-hydroxy-2-(4-hydroxyphenyl)-8-(3-methylbut-2-enyl)chroman-4-one	GU	1
(2R)-7-hydroxy-2-(4-hydroxyphenyl)chroman-4-one	GU	1
Sigmoidin-B	GU	1
(-)-Medicocarpin	GU	1
Eurycarpin A	GU	1
1,3-dihydroxy-8,9-dimethoxy-6-benzofurano[3,2-c]chromenone	GU	1
1,3-dihydroxy-9-methoxy-6-benzofurano[3,2-c]chromenone	GU	1
Glabrone	GU	1
Glabrene	GU	1
Glabranin	GU	1
Glabridin	GU	1
Glyzaglabrin	GU	1
Licopyranocoumarin	GU	1
Liquiritin	GU	1
(E)-3-[3,4-dihydroxy-5-(3-methylbut-2-enyl)phenyl]-1-(2,4-dihydroxyphenyl)prop-2-en-1-one	GU	1
Shinpterocarpin	GU	1
Licoisoflavanone	GU	1
Licoisoflavone B	GU	1
Licoisoflavone	GU	1
Licocoumarone	GU	1
Glycyrin	GU	1
2-(3,4-dihydroxyphenyl)-5,7-dihydroxy-6-(3-methylbut-2-enyl)chromone	GU	1
5,7-dihydroxy-3-(4-methoxyphenyl)-8-(3-methylbut-2-enyl)chromone	GU	1
3-(3,4-dihydroxyphenyl)-5,7-dihydroxy-8-(3-methylbut-2-enyl)chromone	GU	1
Gancaonin B	GU	1
Gancaonin A	GU	1
Licoricone	GU	1
3-(2,4-dihydroxyphenyl)-8-(1,1-dimethylprop-2-enyl)-7-hydroxy-5-methoxy-coumarin	GU	1
Licochalcone G	GU	1
Licochalcone B	GU	1
8-(6-hydroxy-2-benzofuranyl)-2,2-dimethyl-5-chromenol	GU	1
Glypallichalcone	GU	1

Bioactive compound	Herb code	Count
Phaseolinisoflavan	GU	1
Glepidotin B	GU	1
Glepidotin A	GU	1
Semilicoisoflavone B	GU	1
(2S)-6-(2,4-dihydroxyphenyl)-2-(2-hydroxypropan-2-yl)-4-methoxy-2,3-dihydrofuro[3,2-g]chromen-7-one	GU	1
Kanzonols W	GU	1
(E)-1-(2,4-dihydroxyphenyl)-3-(2,2-dimethylchromen-6-yl)prop-2-en-1-one	GU	1
Isotrifoliol	GU	1
Glyasperin C	GU	1
Glyasperin F	GU	1
Glyasperin B	GU	1
Euchrenone	GU	1
(2S)-2-[4-hydroxy-3-(3-methylbut-2-enyl)phenyl]-8,8-dimethyl-2,3-dihydropyrano[2,3-f]chromen-4-one	GU	1
7-Methoxy-2-methyl isoflavone	GU	1
Lupiwighteone	GU	1
Medicarpin	GU	1
Glycyrol	GU	1
DFV	GU	1
Inermine	GU	1
Vestitol	GU	1
Licochalcone a	GU	1
Calycosin	GU	1
Formononetin	GU	1
Jaranol	GU	1
Acacetin	MH	1
Diosmetin	MH	1
Aloe-emodin	MH	1
Eriodictyol	MH	1
Genkwanin	MH	1
Luteolin	MH	1
Linarin	MH	1
Hederagenin	PC	1
(2R)-2-[(3S,5R,10S,13R,14R,16R,17R)-3,16-dihydroxy-4,4,10,13,14-pentamethyl-2,3,5,6,12,15,16,17-octahydro-1H-cyclopenta[a]phenanthren-17-yl]-6-methylhept-5-enoic acid	PC	1
Trametenolic acid	PC	1

Bioactive compound	Herb code	Count
Cerevisterol	PC	1
Ergosta-7,22E-dien-3beta-ol	PC	1
Ergosterol peroxide	PC	1
3beta-Hydroxy-24-methylene-8-lanostene-21-oic acid	PC	1
(+)-catechin	PL	1
Paeoniflorin	PL	1
Paeoniflorgenone	PL	1
(3S,5R,8R,9R,10S,14S)-3,17-dihydroxy-4,4,8,10,14-pentamethyl-2,3,5,6,7,9-hexahydro-1H-cyclopenta[a]phenanthrene-15,16-dione	PL	1
6-methylgingediacetate2	ZO	1
Poriferast-5-en-3beta-ol	ZO	1
Dihydrocapsaicin	ZO	1

Table S4. Differential expression analysis of the 16 core genes based on GEO datasets

Gene symbol	Protein name	Log_2FC	P-Value
TGFB1	Transforming growth factor beta-1	-2.354	< 0.001
GJA1	Gap junction alpha-1 protein	-4.862	< 0.001
CYP1A2	Cytochrome P450 1A2	7.774	< 0.001
CDK1	Cyclin-dependent kinase 1	-3.444	< 0.001
EGF	Pro-epidermal growth factor	-2.646	< 0.001
NR1I3	Nuclear receptor subfamily 1 group I member 3	2.401	< 0.001
E2F2	Transcription factor E2F2	-2.431	< 0.001
NR1I2	Nuclear receptor subfamily 1 group I member 2	2.792	< 0.001
CYP2C19	Cytochrome P450 2C19	2.164	< 0.001
CYP3A4	Cytochrome P450 3A4	2.242	< 0.001
PDE5A	cGMP-specific 3′,5′-cyclic phosphodiesterase	-2.543	< 0.001
ADH1B	Alcohol dehydrogenase 1B (class I), beta polypeptide	4.052	< 0.001
F3	Tissue factor	-2.387	< 0.001
ALOX5	Arachidonate 5-lipoxygenase	-2.021	< 0.001
CES1	Carboxylesterase 1	3.482	< 0.001
IGF2	Insulin-like growth factor II	2.872	< 0.01

Table S5. Molecular docking of representative XYS compounds with hub target proteins, together with the corresponding protein structures and docking grid box parameters.

Target protein	PDB ID	Bioactive compound	Affinity (kcal/mol)	Grid Box Center (x, y, z) (Å)	Grid Box Size (x, y, z) (Å)
ALOX5	3V92	Beta-sitosterol	**-7.18**	(-10.218, -66.251, -32.777)	114.8 × 114.8 × 114.8
		Kempferol	-5.43		107.8 × 107.8 × 107.8
		Luteolin	-6.11		110.95 × 110.95 × 110.95
		Quercetin	-5.02		123.90 × 123.90 × 123.90
CYP1A2	2HI4	Beta-sitosterol	**-7.92**	(7.330, 21.536, 25.282)	88.55 × 88.55 × 88.55
		Kempferol	-6.73		87.15 × 87.15 × 87.15
		Luteolin	-6.72		89.95 × 89.95 × 89.95
		Quercetin	**-7.24**		73.15 × 73.15 × 73.15
CYP3A4	4NY4	Beta-sitosterol	**-8.66**	(-19.675, -24.262, -14.069)	90.3 × 90.3 × 90.3
		Kempferol	-6.65		83.65 × 83.65 × 83.65
		Luteolin	-6.14		89.25 × 89.25 × 89.25
		Quercetin	-6.01		92.05 × 92.05 × 92.05
F3	8QOD	Beta-sitosterol	-6.23	(29.545, 16.444, 59.183)	91.35 × 91.35 × 91.35
		Kempferol	-5.39		88.55 × 88.55 × 88.55
		Luteolin	-5.87		84.35 × 84.35 × 84.35
		Quercetin	-5.49		78.05 × 78.05 × 78.05
PDE5A	6VBI	Beta-sitosterol	**-6.93**	(8.704, 31.454, 139.902)	102.9 × 102.9 × 102.9
		Kempferol	**-6.85**		117.95 × 117.95 × 117.95
		Luteolin	**-7.11**		113.75 × 113.75 × 113.75
		Quercetin	**-7.02**		107.10 × 107.10 × 107.10

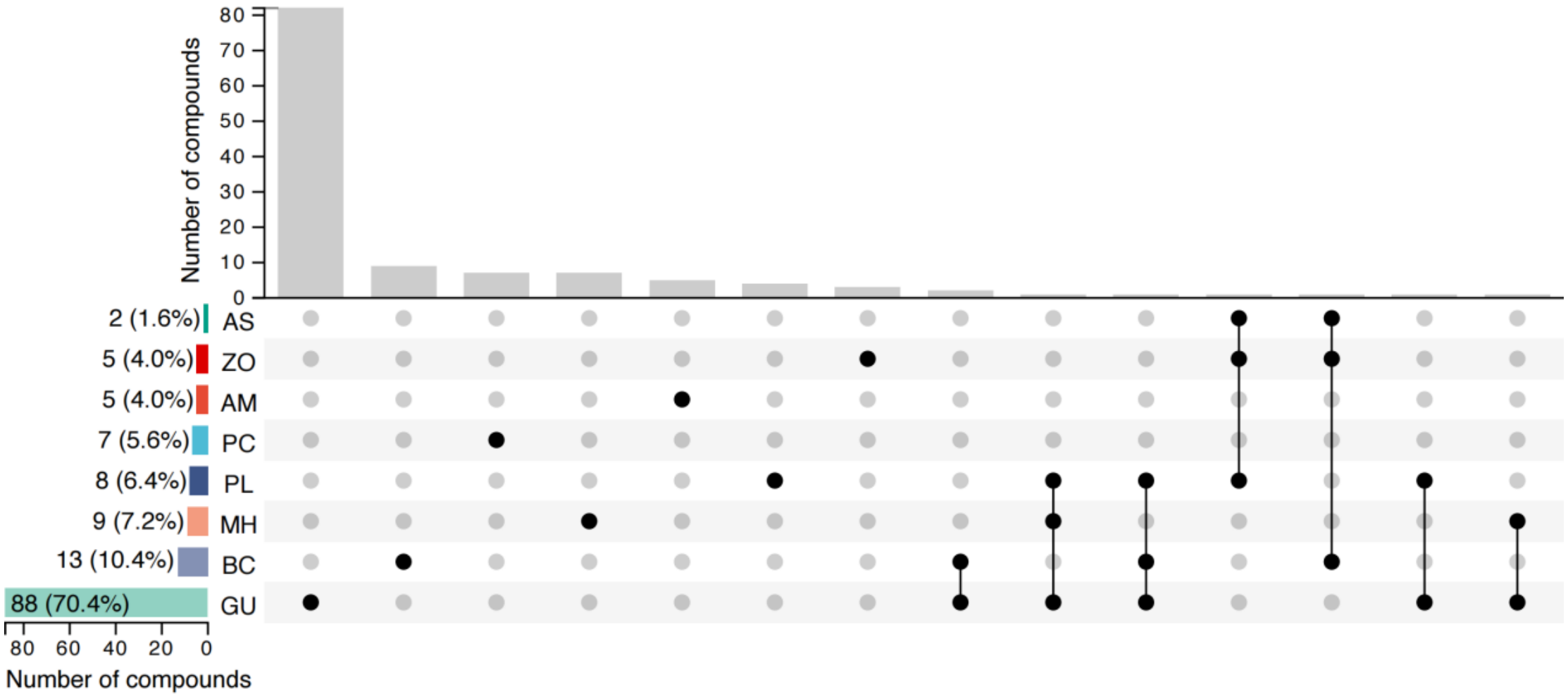

Figure S1. Distribution and overlap of active compounds across herbal components in the Xiao-Yao-San (XYS). The UpSet plot shows the distribution patterns of 125 active compounds among the eight herbs. The horizontal bar chart (left) displays the total number of compounds in each herb. The vertical bars represent the number of compounds in each specific herb combination, with the leftmost bar showing GU-specific compounds and smaller bars showing compounds shared among different herb combinations. Most compounds are herb-specific, with limited overlap between herbs. Herb codes are defined in Table S1.

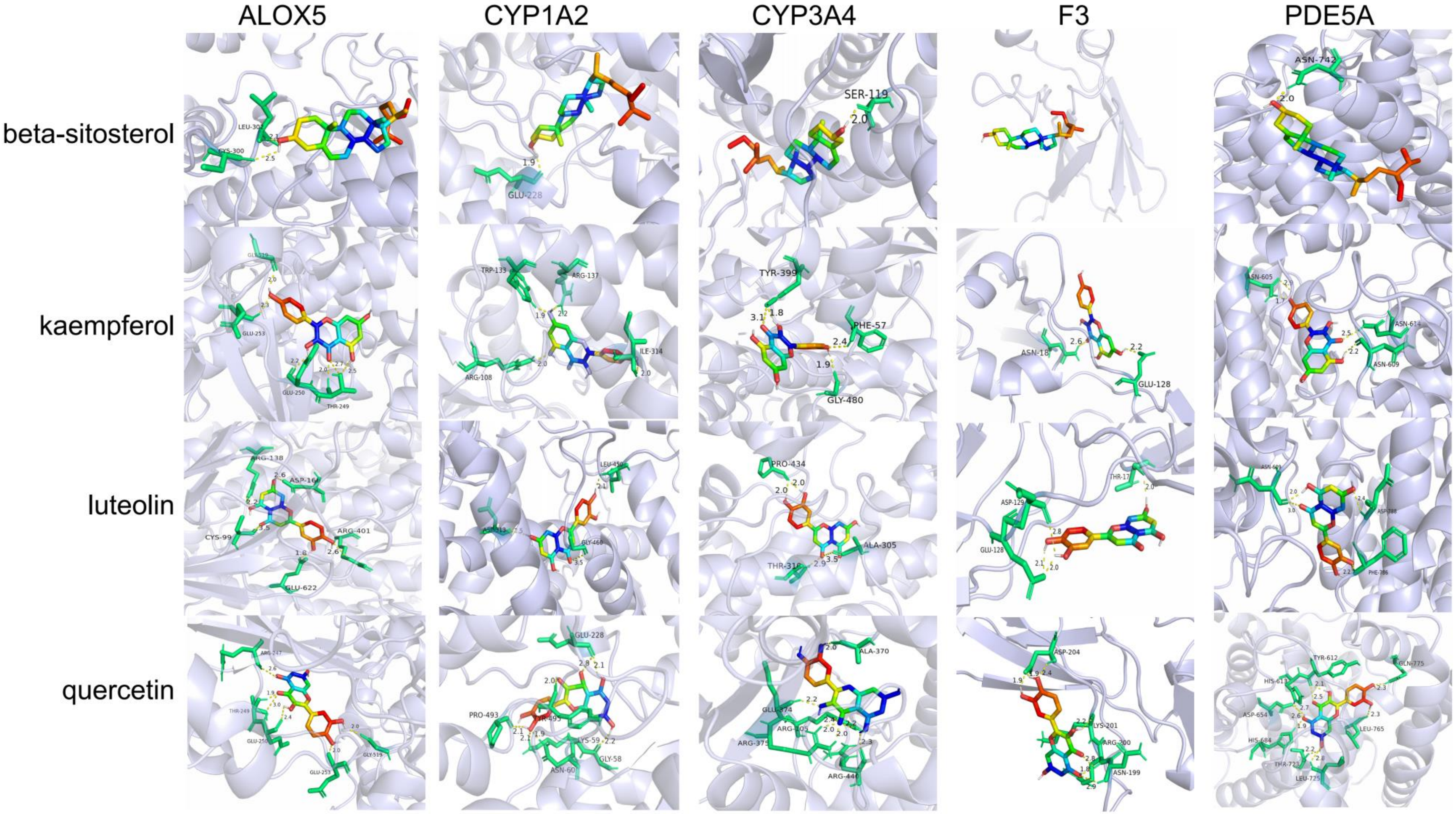

Figure S2. Enlarged views of the predicted docking poses between the potential hub targets and four core compounds, highlighting the binding pocket residues involved in putative interactions (green sticks), ligands (colored sticks), and predicted hydrogen bonds (yellow dashed lines).
